# Unsupervised Machine Learning Reveals a Vulvodynia-Predominant Subtype in Bladder Pain Syndrome/Interstitial Cystitis

**DOI:** 10.7759/cureus.62585

**Published:** 2024-06-18

**Authors:** Nobuo Okui

**Affiliations:** 1 Dentistry, Kanagawa Dental University, Yokosuka, JPN

**Keywords:** overactive bladder symptom score, overactive bladder questionnaire short form, pelvic pain and urgency/frequency scores, interstitial cystitis problem index, interstitial cystitis symptom index, vulvodynia swab test, numerical rating scale-11, vulvodynia, interstitial cystitis, bladder pain syndrome

## Abstract

Background

Bladder pain syndrome/interstitial cystitis (BPS/IC) is a chronic condition characterized by pelvic pain and urinary symptoms. Despite its significant impact on patients’ quality of life, the heterogeneity of BPS/IC symptoms and the presence of comorbidities such as vulvodynia may not be adequately captured by validated questionnaires. Identifying vulvodynia in BPS/IC patients is crucial for providing appropriate treatment options. This study aimed to identify subtypes of BPS/IC patients using unsupervised machine learning and to investigate the prevalence of vulvodynia in each subtype.

Methods

We conducted a prospective cross-sectional study of 123 BPS/IC patients and 64 age-matched controls. Hierarchical clustering was performed using data from validated questionnaires, including the Numerical Rating Scale-11, Interstitial Cystitis Symptom Index (ICSI), Interstitial Cystitis Problem Index (ICPI), Pelvic Pain and Urgency/Frequency scores, Overactive Bladder Questionnaire Short Form (OABq SF), Overactive Bladder Symptom Score (OABSS), and Pelvic Floor Distress Inventory-20. The optimal number of clusters was determined using the elbow method, and the characteristics of each cluster were analyzed. All participants underwent a vulvodynia swab test to assess vulvodynia symptoms.

Results

Unsupervised machine learning revealed three distinct clusters of BPS/IC patients. Clusters 0 and 2 differed significantly, with Cluster 2 characterized by significantly higher vulvodynia scores compared to other clusters (P < 0.001). In contrast, Cluster 2 had lower bladder pain scores (ICSI and ICPI) and overactive bladder symptom scores (OABq SF and OABSS) compared to other clusters. Clusters 0 and 1 were characterized by a predominance of bladder pain and urinary frequency symptoms, with Cluster 0 exhibiting more severe symptoms.

Conclusions

Our study identified distinct subtypes of BPS/IC patients using unsupervised machine learning, with Cluster 2 representing a vulvodynia-predominant subtype. This finding, along with the potential of targeted therapies such as non-ablative erbium YAG laser for vulvodynia, underscores the importance of assessing extravesical symptoms, particularly vulvodynia, for the diagnosis and treatment of BPS/IC. A tailored approach, including laser therapy for vulvodynia-predominant patients, may be necessary for optimal management of BPS/IC. The vulvodynia swab test plays a crucial role in assessing vulvodynia symptoms, underlining the limitations of validated questionnaires in capturing the full spectrum of BPS/IC symptoms. A comprehensive evaluation of patients, including the vulvodynia swab test, is essential for accurate subtyping and management of BPS/IC. Further research with larger sample sizes and investigation of the relationship between identified subtypes and other clinical data is warranted to advance our understanding and management of BPS/IC.

## Introduction

Bladder pain syndrome/interstitial cystitis (BPS/IC) is a chronic condition characterized by pelvic pain and urinary symptoms that significantly affect patients’ quality of life [1,2]. The etiology of BPS/IC is multifactorial, and the condition often presents with various comorbidities, such as pelvic organ inflammation, vulvodynia, and pelvic floor myofascial pain, which have been reported to be highly prevalent [3-6]. Furthermore, inflammation in the BPS/IC may extend to other pelvic organs, potentially contributing to the complex symptomatology of this condition [7,8].

Among these comorbidities, vulvodynia has gained attention in recent years. Reports suggest that 30-50% of BPS/IC patients have comorbid vulvodynia [4], indicating that vulvodynia may be a systemic symptom of BPS/IC. Several studies have demonstrated that laser treatment for vulvodynia can lead to an improvement in BPS/IC symptoms [9,10], suggesting that vulvodynia may play a significant role in the pathophysiology of BPS/IC and could be a potential therapeutic target. Okui et al. reported a case series showing the efficacy of vaginal erbium laser (VEL) treatment (RenovaLase, SP Dynamis, Fotona d.o.o, Ljubljana, Slovenia) for patients with both vulvodynia and BPS/IC [10].

Despite the importance of vulvodynia in BPS/IC patients, it may not be adequately assessed in clinical practice [3,4]. Validated questionnaires are commonly used to evaluate BPS/IC symptoms, but they may not fully capture extravesical symptoms, such as vulvodynia [9,10]. This limitation could contribute to suboptimal treatment outcomes, and addressing vulvar health may be crucial for improving symptom management and quality of life [10].

In this study, we aimed to perform phenotypic clustering of BPS/IC patients using validated questionnaires and investigate the prevalence of vulvodynia in each cluster. In doing so, we sought to determine whether vulvodynia could be predicted solely based on the results of these questionnaires. This information could contribute to the development of more targeted diagnostic and treatment strategies for BPS/IC patients with comorbid vulvodynia.

## Materials and methods

Study design and participants

This was a prospective cross-sectional study of patients who presented with pelvic pain for at least three months. The study protocol was approved by the Regional Ethics Committee of Kanagawa Dental University in 2023 (approval number 959). This study was registered in the UMIN-CTR and was conducted in accordance with the principles of the Declaration of Helsinki. Participants were fully informed of the study's purpose and methods and provided written consent. The data were anonymized and reported only as aggregate results. This study recruited patients from the Yokosuka Urogynecology and Urology Clinic (Yokosuka, Japan) and conducted statistical analyses at Kanagawa Dental University (Yokosuka, Japan) between September 2023 and April 2024.

Given the previous literature, study duration, and relatively rare nature of the condition, we estimated that a sample size of 123 patients would be appropriate. This sample size was deemed sufficient to maintain 80% power and detect specific effect sizes based on statistical calculations.

Diagnosis and inclusion/exclusion criteria

The study population consisted of patients presenting with pelvic pain for at least three months due to BPS/IC. Patients were recruited through random selection or consecutive sampling from those who visited the clinic during the specified study period. A BPS/IC diagnosis was made by specialists based on a detailed medical history, a physical examination, and the application of high-sensitivity diagnostic criteria. According to these criteria, the possibility of BPS/IC was considered if there was pain, pressure, or discomfort in the pelvic region, along with daytime urinary frequency (≥10 times), pain, pressure, or discomfort-related urgency.

The validated questionnaires were self-administered by patients in a private setting with the assistance of female nurses due to the inclusion of sexual function items. Patients with Pelvic Pain and Urgency/Frequency (PUF) scores ≥5 were enrolled in the study [3]. PUF scores assessed chronic pelvic pain, urgency, and urinary frequency.

Exclusion criteria included other urological or gynecological diagnoses that could explain the urinary symptoms or pain (e.g., overactive bladder, recurrent urinary tract infections, urinary retention, voiding dysfunction, uterine/ovarian cancer, uterine infection, vaginal pessary use, and pelvic organ prolapse stage ≥3), history of invasive treatment, pregnancy, diabetes, neurological or rheumatic diseases, and current smoking.

Patients with Hunner’s lesions were considered separate disease entities and were excluded from the analysis.

Polycystic ovary syndrome (PCOS) was confirmed using ultrasound in the study population.

The control group consisted of asymptomatic women (undergoing cervical cancer screening) with a PUF score of <4, matched for age.

Assessments

After obtaining informed consent, the following validated questionnaires were administered: Numerical Rating Scale-11 (NRS-11) [11], Vulvodynia Swab Test [12], Interstitial Cystitis Symptom Index (ICSI) [13], Interstitial Cystitis Problem Index (ICPI) [14], PUF scores [3], Overactive Bladder Questionnaire Short Form (OABq SF) [15], Overactive Bladder Symptom Score (OABSS) [16], and Pelvic Floor Distress Inventory-20 (PFDI-20) [17]. The NRS-11 measures pain intensity, the Vulvodynia Swab Test assesses vulvar sensitivity, the ICSI and ICPI evaluate interstitial cystitis symptoms and their impact on daily life, the PUF scores assess urinary urgency and frequency, the OABq SF and OABSS evaluate overactive bladder symptoms, and the PFDI-20 assesses pelvic floor disorders. The Female Sexual Function Index (FSFI), which is used to assess sexual function, was not included in the clustering analysis. This is because the FSFI is not commonly used for the diagnosis of BPS/IC, and the primary objective of this study was to investigate the presence of vulvodynia within clusters based on the standard questionnaires for BPS/IC, such as ICSI, ICPI, and PUF. Including the FSFI in the analysis could potentially bias the clustering results toward the presence of vulvodynia. Additionally, there were concerns about the reliability of the FSFI data due to the high number of missing values. Future studies may consider incorporating sexual function assessments to provide a more comprehensive understanding of BPS/IC patients. The Vulvodynia Swab Test was performed at the 2, 4, 8, and 10 o’clock positions on the vaginal vestibule, and the level of pain at each site was evaluated using a visual analog scale (0, no pain; 10, worst pain). The total scores from the four sites were used in the analysis.

The distribution of PUF scores in the BPS/IC patient and control groups was compared using histograms and kernel density estimations (KDEs) [18]. KDE is a nonparametric method for estimating the probability density function of a random variable. Histograms were constructed using 20 bins, and KDE curves were generated by optimizing the smoothing parameter.

Hierarchical clustering based on validated BPS/IC questionnaires and vulvodynia scores was performed to assess the symptom profiles of the patient groups [19].

First, a distance matrix between patients was calculated using multiple standardized clinical indicators, with the Euclidean distance used to quantify the similarity of symptoms. Hierarchical clustering was then performed using Ward’s method, and a dendrogram was created [20].

The elbow method is used to determine the optimal number of clusters. The sum of squared errors (SSE) within clusters was calculated for different cluster numbers, and the point at which the decrease in SSE slowed was identified as the optimal number of clusters [21]. K-means clustering was then used to assign patients to the identified clusters.

Exploratory analysis of patient groups was conducted using principal component analysis (PCA) to visualize high-dimensional datasets [22]. The validity of clustering was further evaluated using the elbow method and K-means clustering, which are based on discrete mathematical optimization techniques [23].

Statistical analysis

Descriptive statistics were used to summarize baseline demographic and clinical characteristics. Continuous variables are presented as mean ± standard deviation, and categorical variables are reported as counts (%).

The Kruskal-Wallis test was performed to assess the statistical differences among the three clusters for each indicator, followed by post hoc multiple comparisons using the Tukey-Kramer method. A one-way ANOVA was used to evaluate the differences in vulvodynia scores between clusters.

Statistical analyses were performed using Python packages including Scipy, NumPy, Pandas, Matplotlib, Seaborn, Pingouin, and Scikit-learn.

## Results

Patient characteristics

From September 2023 to the end of April 2024, 348 patients with complaints of urogenital pain visited our clinic. A total of 217 patients (63.8%) were excluded, including 38 with urinary tract infections, five with diabetic neuropathy, two with herpes zoster, four with malignant bladder tumors, 16 with ureteral stones, 112 with uterine prolapse, 25 with mesh pain from previous POP surgery, and 15 with a history of pelvic pain lasting less than three months. The remaining 131 patients (37.6%) complained of pelvic pain lasting three months or longer, scored 5 or higher on the PUF scores, and met the American Urological Association guidelines for BPS/IC; thus, they were classified into the BPS/IC group. Additionally, eight patients were found to have Hunner’s lesions on cystoscopy and were excluded from the current sampling. A total of 123 patients were included in this study. From the patients who underwent cervical cancer screening, 64 age-matched individuals with PUF scores of 4 or lower were selected as the control group. Table 1 shows the patients’ characteristics.

**Table 1:** Demographic and clinical characteristics of BPS/IC patients compared to control subjects P-values were calculated using a two-tailed t-test for independent samples. BPS/IC, bladder pain syndrome/interstitial cystitis; GERD, gastroesophageal reflux disease; IBS, irritable bowel syndrome; PCOS, polycystic ovary syndrome

Variable	BPS/IC	Controls	P-value
Group count	123	64	N/A
Age: years (mean ± SD)	49.44 ± 11.75	55.38 ± 12.43	0.0015
BMI: kg/m² (mean ± SD)	23.50 ± 2.61	23.35 ± 2.85	0.7163
Hormonal birth control: percent (n)	6.5% (8)	1.6% (1)	N/A
Anxiety	27.6% (34)	4.7% (3)	0
Depression	11.4% (14)	6.2% (4)	0.39
Endometriosis	2.4% (3)	0.0% (0)	0.55
Fibromyalgia	2.4% (3)	0.0% (0)	0.55
GERD	0.8% (1)	3.1% (2)	0.27
Hyperlipidemia	17.9% (22)	20.3% (13)	0.84
Hypertension	11.4% (14)	21.9% (14)	0.09
IBS	13.0% (16)	1.6% (1)	0.02
Migraine	7.3% (9)	3.1% (2)	0.41
Nephrolithiasis	1.6% (2)	9.4% (6)	0.04
PCOS	1.6% (2)	3.1% (2)	0.61

**Figure 1:**
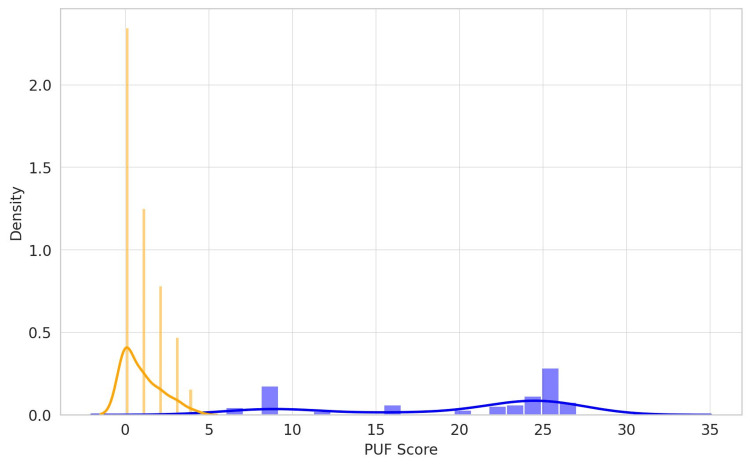
Distribution of PUF scores The vertical axis represents the normalized density of patients across the range of PUF scores, shown both as a histogram and a KDE. The horizontal axis indicates the PUF scores, ranging from zero to the highest score in the dataset. The orange bars represent the histogram for the control group, showing the frequency of PUF scores, whereas the orange curve is the KDE, depicting a smooth estimate of the shape of the distribution. Similarly, the blue bars and corresponding blue curves represent the histogram and KDE, respectively, for the BPS/IC patient group. BPS/IC, bladder pain syndrome/interstitial cystitis; KDE, kernel density estimation; PUF, Pelvic Pain and Urgency/Frequency

Figure 1 shows the distribution of PUF scores in the control group, represented by an orange bar graph, where the shape of the PUF score distribution using the kernel is depicted as an orange curve. On the other hand, the blue bar graph represents the distribution of PUF scores in the BPS/IC patient group, and the blue curve shows the shape of the distribution in the same patient group using KDE. These graphs visually demonstrate a substantial difference in PUF scores between the BPS/IC patient and control groups, with the control group generally exhibiting lower PUF scores. This suggests that sampling using PUF scores was performed appropriately.

Clustering

**Figure 2:**
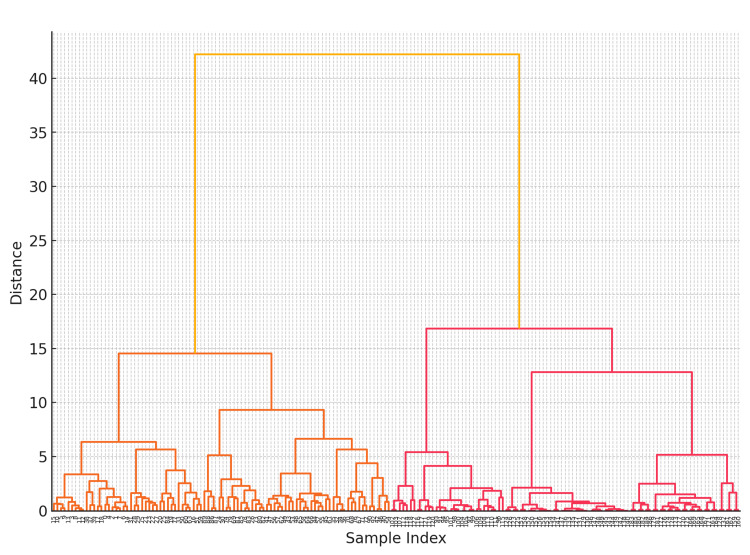
Hierarchical clustering dendrogram This dendrogram is constructed based on the similarity between the BPS/IC patient samples, with the vertical axis showing the merging distance required to form each cluster and the horizontal axis representing individual patient samples. BPS/IC, bladder pain syndrome/interstitial cystitis

Figure 2 shows the dendrogram created to visualize the results of hierarchical clustering calculated using validated questionnaires (NRS-11 [11], ICSI [13], ICPI [14], PUF scores, OABq SF [15], OABSS [16], and PFDI-20 [17]) as variables in the BPS/IC group. This dendrogram was constructed based on the distances between the data points of BPS/IC patients, showing that patients with similar symptom profiles are clustered together at low distances. The vertical axis in the figure represents the merging distance in the clustering, whereas the horizontal axis represents each patient. As the merging distance increased, the patients with dissimilar symptom profiles were integrated.

**Figure 3:**
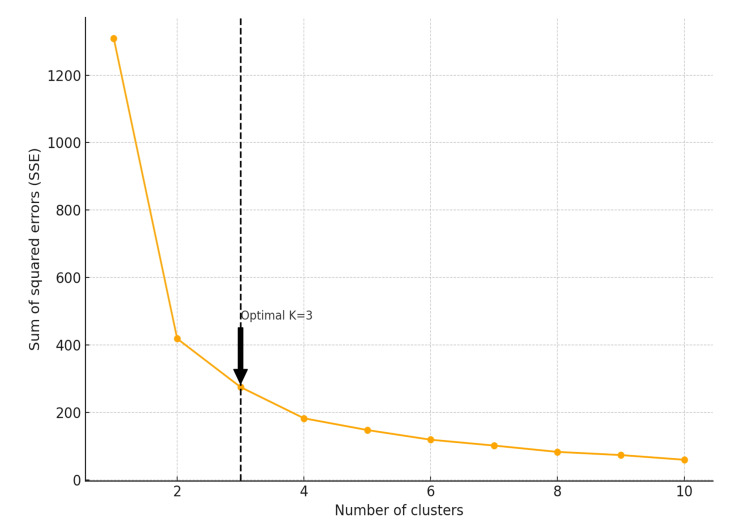
Optimization of the number of clusters using the elbow method The y-axis represents the SSE and the x-axis represents the number of clusters. The dashed vertical line at K = 3 indicates the optimal number of clusters, where the rate of decrease in SSE slows down significantly. SSE, sum of squared errors

Figure 3 shows the results of the analysis using the elbow method to optimize the number of clusters. This method involves plotting SSE within clusters as a function of the number of clusters and identifying the “elbow” point where SSE decreases sharply. Based on the elbow method plot, the number of clusters was determined to be three, where SSE first decreased significantly, and then the rate of decrease slowed down. This is clearly shown in the graph, where the decrease in the SSE gradually increases as the number of clusters increases from two to three. Based on the results of the elbow analysis, the optimal number of clusters for the dataset was determined to be three. The SSE reduction curve showed an inflection point at an increase from two to three clusters, where the decrease in the SSE became gradual.

**Figure 4:**
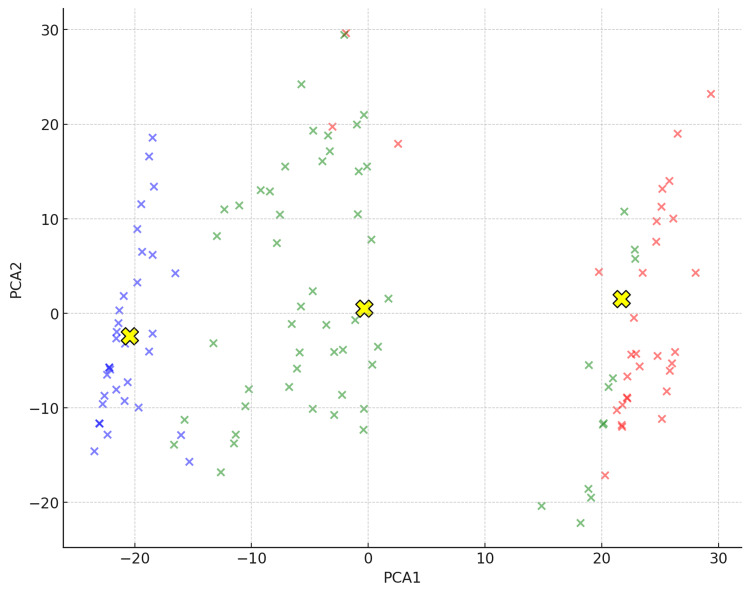
Centroid plot of three clusters using PCA PCA1 and PCA2 are the principal components that capture the maximum variance in the data, visually distinguishing the symptom profiles of each cluster. Cluster 0 is represented in red, Cluster 1 in green, and Cluster 2 in blue. The y-axis represents PCA2, and the x-axis represents PCA1. The yellow “X” marks indicate the centroids of the clusters, capturing the central points of each cluster in the reduced dimensional space. PCA, principal component analysis

Figure 4 shows the results of the k-means clustering. In the centroid plot, the patient groups are clearly distinguishable by their colors, with Cluster 0 in red, Cluster 1 in green, and Cluster 2 in blue. They were positioned on the plane of the first principal component (PCA 1) and the second principal component (PCA 2) obtained by PCA. These results support the idea that Clusters 0, 1, and 2 are clearly distinguishable subgroups, each representing different symptom profiles.

Proportion of vulvodynia and other characteristics in each cluster

**Figure 5:**
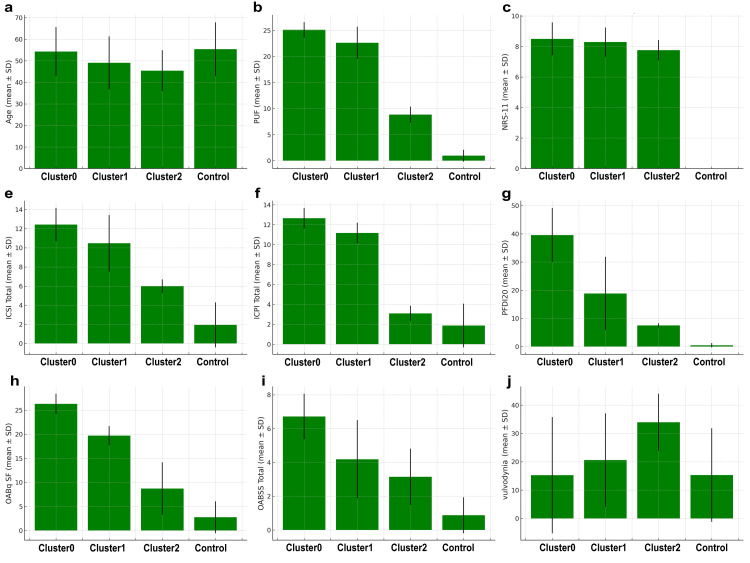
Data of each indicator in clusters and control group The mean values and standard deviations of nine indicators (age, NRS-11, ICSI, ICPI, PUF, OABq SF, OABSS, PFDI20, and vulvar swab test), for subjects in each cluster (0, 1, and 2) and the control group are displayed in the bar graph. Each bar represents the mean value of an indicator, and the error bars indicate the standard deviations. The y-axis represents age (years) and the points for each score, while the x-axis represents each cluster (0, 1, and 2) and the control group.

Figure 5 shows nine indicators for subjects divided into Clusters 0, 1, and 2, and a control group. The indicators are as follows: (a) age; (b) NRS-11; (c) Vulvodynia swab test; (d) ICSI; (e) ICPI; (f) PUF scores; (g) OABq SF; (h) OABSS; and (i) PFDI20.

To assess statistical differences among the three clusters, the Kruskal-Wallis test was performed for each indicator: age, NRS-11, vulvodynia score, ICSI, ICPI, PUF scores, OABq SF, OABSS, and PFDI20. Post hoc multiple comparisons were conducted using the Tukey-Kramer method.

The Kruskal-Wallis test revealed statistically significant differences for all indicators among the clusters (all P < 0.001).

The distribution of vulvodynia scores is particularly noteworthy. While the vulvodynia scores in Cluster 0 were similar to those in the control group (cluster 0:15.27 ± 20.48, control: 15.33 ± 16.52), they were significantly higher in Cluster 2 (33.94 ± 10.11, P = 2.20 × ^10-6^. Furthermore, when the differences in vulvodynia scores between clusters were evaluated using one-way ANOVA, a statistically significant difference was confirmed (F = 10.63, P = 1.80 × 10^-6^).

On the other hand, the OABq SF (8.73 ± 5.45) and OABSS (3.15 ± 1.66) scores in Cluster 2 were comparable to those in the control group (OABq SF: 2.75 ± 3.30, OABSS: 0.88 ± 1.06), and the bladder pain assessed by ICSI and ICPI was lower compared to the other clusters (ICSI: Cluster 0: 12.44 ± 1.74, Cluster 1: 10.48 ± 2.95, Cluster 2: 6.00 ± 0.71; ICPI: Cluster 0: 12.66 ± 1.04, Cluster 1: 11.17 ± 1.03, Cluster 2: 3.09 ± 0.77).

## Discussion

In this study, we performed clustering analysis using standardized questionnaires and were able to classify BPS/IC patients into three clusters. This finding is consistent with the results of previous studies, suggesting that BPS/IC symptoms are diverse. In particular, Cluster 2 showed different characteristics from the other clusters, with extravesical symptoms, especially vulvar pain, being predominant. This is consistent with the results of a previous study by Mwesigwa et al., who also characterized Group 1 as having frequency and pain related to the voiding cycle, Group 2 as having fluctuating pelvic discomfort and straining during urination, urgency with frequency (no incontinence), and a feeling of residual urine (no retention), and Group 3 as having constant urethral and vaginal pain unrelated to voiding [23]. This result suggests that defining more homogeneous patient subgroups could lead to improved care [23]. However, the authors did not investigate the presence of vulvodynia.

In Cluster 2, the vulvodynia swab test results were significantly higher than those of the other clusters and the control group. In contrast, the ICSI, ICPI, OABq SF, and OABSS scores related to bladder pain and overactive bladder were milder than in the other clusters, although there were more symptoms than in the control group. These results suggest that BPS/IC has at least one bladder symptom-predominant subtype (Cluster 0) and a vulvar symptom-predominant subtype (Cluster 2). This was the major finding of the present study.

The identification of the vulvodynia-predominant subtype may contribute to the understanding of the pathophysiology of BPS/IC and the optimization of treatment strategies. In fact, it is known that treating vulvodynia in BPS/IC patients improves bladder symptoms. Gardella et al. reported that vulvodynia treatment with local estrogen therapy alleviated BPS/IC symptoms [24]. Okui et al. reported that in a group of refractory cases that did not respond to any approach to the bladder, VEL treatment to improve vaginal health status resulted in an improvement in bladder pain symptoms [25]. Butrick et al. reported an improvement in BPS/IC symptoms in cases in which vaginal health status improved with transvaginal photobiomodulation [26]. Okui et al. focused on vulvodynia and reported that combined vaginal erbium/neodymium laser therapy (VEL + Nd:YAG) was highly effective for BPS/IC symptoms [27]. In a case report by Okui et al., they pathologically examined the vaginal and bladder mucosa and found improvements after VEL + Nd:YAG treatment in a case with severe BPS/IC and vulvodynia [28]. The interaction between BPS/IC and vulvodynia was highlighted. Therefore, it is predicted that this subtype of patient will require an approach that is different from conventional bladder-centric treatments [28].

However, the results of this study suggest that it may be difficult to confirm whether a patient is in Cluster 2 and to capture the presence of vulvodynia using only standardized questionnaires in clinical practice. A comprehensive analysis of subjective and objective data revealed the diversity of BPS/IC, the importance of extravesical symptoms, and the confirmation of vulvodynia is very important. Guidelines in each country emphasize the need to incorporate a more comprehensive evaluation of patients [1,28-30].

The limitations of this study include its small sample size and the fact that it was a single-center study. It is expected that the findings of this study will be further developed by examining their relevance to other clinical data. In addition, it is important to develop individualized treatment strategies based on the subtypes identified in this study, and there is a risk of biased information in the clustering and confirmation of vulvodynia. BPS/IC is a complex disease, and a multifaceted approach is essential for its diagnosis and treatment. The results of this study are expected to contribute to deepening the understanding of BPS/IC and developing new treatment strategies that lead to improved QOL in patients. In this study, following previous research, we included patients with PCOS in the BPS/IC population. However, the impact of PCOS on PUF remains unclear, and evidence showing a positive correlation between PCOS and PUF is limited [31]. To better represent the reality of BPS/IC, we deliberately included patients with PCOS. Nevertheless, future studies may change the perspective on whether PCOS should be included in the clustering of BPS/IC. The FSFI was not included in the clustering analysis as it is not commonly used in the diagnosis of BPS/IC. The primary objective was to investigate the presence of vulvodynia within clusters based on standard BPS/IC questionnaires. Including the FSFI could have potentially biased the results, and there were concerns about data reliability due to missing values. Future studies may consider incorporating sexual function assessments.

## Conclusions

This study used unsupervised machine learning to successfully classify BPS/IC patients into three distinct subtypes. Cluster 2 was identified as a vulvodynia-predominant subtype, which, coupled with recent evidence supporting the efficacy of non-ablative erbium YAG laser therapy for vulvodynia, highlights the importance of assessing extravesical symptoms, particularly vulvodynia, in the diagnosis and treatment of BPS/IC. A tailored treatment approach, including laser therapy, may be necessary for vulvodynia-predominant patients. The vulvodynia swab test plays a crucial role in assessing vulvodynia, underscoring the limitations of standardized questionnaires. A comprehensive evaluation of patients, including the vulvodynia swab test, is essential for accurate subtyping and management of BPS/IC. Further research with larger sample sizes and investigation of the relationship between identified subtypes and other clinical data is warranted to advance our understanding and management of BPS/IC.
